# HRQoL in Barth Syndrome: Agreement between Child Self-reports and Parent Proxy-reports and Its Relationship to Parental HRQoL

**DOI:** 10.29245/2690-0009/2019/2.1104

**Published:** 2019-06-13

**Authors:** Yoonjeong Lim, Consuelo M. Kreider, Mary Alvarez, Roxanna M. Bendixen

**Affiliations:** 1 Department of Occupational Therapy Byrdine F. Lewis College of Nursing & Health Professions, Georgia State University, Atlanta, GA, USA; 2 Department of Occupational Therapy, College of Public Health and Health Professions, University of Florida, Gainesville, FL, USA; 3 Department of Occupational Therapy, School of Health and Rehabilitation Sciences, University of Pittsburgh, Pittsburgh, PA, USA

**Keywords:** Barth síndrome, Health-related quality of life, HRQoL, Proxy-report, Rare Diseases, Self-report

## Abstract

**Purpose:**

Barth syndrome is an X-linked rare disorder that typically affects only males. This study investigates 1) agreement between child self-reports and parent proxy-reports of HRQoL in boys with Barth syndrome and 2) relationship between parental HRQoL and parent proxy-reports of HRQoL for the child.

**Materials and methods:**

Twenty-eight boys with Barth syndrome and their parents participated in this study. The PedsQL™ 4.0 and the PedsQL™ Family Impact Module were used to measure HRQoL of the boys, and the parents’ HRQoL, respectively. The Intraclass Correlation Coefficient was used to test agreement between the child self-reports and parent proxy-reports of HRQoL. The Spearman correlation coefficient was used to test the relationship between parental HRQoL and parent proxy-reports of HRQoL for the child.

**Results:**

The agreement between the child self-reports and the parent proxy-reports showed moderate-to-good agreement. Higher parental HRQoL was significantly related to higher ratings of the parents on their children’s HRQoL (*p* < .05).

**Conclusions:**

This study broadens understanding of HRQoL of boys with Barth syndrome using both child self-reports and parent proxy-reports. The findings indicate that the parent proxy-report of HRQoL should be used in conjunction with the child self-report when making client-centered health decisions.

## Introduction

Barth syndrome (BTHS) is a rare, life-threatening X-linked genetic disorder that typically affects only males^1^. First described in 1983, BTHS has been diagnosed in fewer than 500 living males worldwide; however, evidence suggests that BTHS may be significantly underdiagnosed due to the wide variety of clinical features associated with the disorder^2^. Therefore, the exact prevalence of BTHS remains unknown, though the disorder is estimated to occur in roughly 1 out of every 300,000 – 400,000 U.S. births^2,3^. Once regarded as primarily a cardiac condition, BTHS is now recognized as a multi-system disorder caused by a complex inborn metabolic error^1,2,4^.

While the exact nature and degree of symptoms vary considerably in different individuals, cardinal characteristics of BTHS include cardiomyopathy, neutropenia, muscle hypoplasia and weakness, growth delays and short stature, exercise intolerance, 3-methylglutaconic aciduria, and cardiolipin deficiency ^5,6^. These symptoms are typically detected in infancy or early childhood, though diagnosis of BTHS is often difficult or delayed^3,7^. Though survival rates have improved significantly in recent years, individuals with BTHS remain predisposed to premature death due to cardiac issues and risk of infection related to neutropenia^5,8^. BTHS is frequently associated with physical effects, such as fatigue, pain, and feeding problems, as well as mild cognitive effects on visuo-spatial skills and motor learning^7,9,10,11,12^. These cognitive and physical effects may contribute to increased functional difficulties in academics, prevocational/vocational activities, and independent living skills^13^. In addition, boys with BTHS have been reported to have difficulty getting along with peers, often experiencing bullying because of their small stature and limits on physical exertion^11,14^. These peer interactions contribute to increased stress and may lead to lowered self-esteem^14^.

Given the impact of BTHS on an individual’s functioning and well-being, it is understandable that initial research indicates that boys with BTHS have significantly reduced quality of life as related to their health^11^. Health-related quality of life (HRQoL) is a multidimensional construct that considers the impact of disease or health conditions on overall health and daily living^15,16^. Physical, emotional, and social functioning are considered when assessing HRQoL^16^. When a child can self-report his or her health status, child self-report is typically used^17^. However, if the child is very young or cannot complete the self-report due to illness or cognitive impairment, parents may be asked to report on their child’s HRQoL via parent proxy-report^18,19^. While some earlier studies misguidedly attempted to determine whose report was more “accurate”, current research typically accepts that both the parent and child reports provide invaluable information and that comprehensive evaluation should include consideration of both the child and parent perspectives^17^.

Previous studies of children with chronic conditions report varying degrees of agreement between child self-reports and parent proxy-reports of HRQoL^20,21,22^. However, in general, parents of children with chronic conditions typically report lower HRQoL for the children than the children themselves^23,24,25,26^. This contrasts with parents of healthy children, who tend to rate their children’s HRQoL higher than the children themselves^15,17^. By identifying which aspects of HRQoL show the greatest discrepancies in the level of agreement between child self-reports and parent proxy-reports of HRQoL, clinicians can better understand the differences in perspectives of HRQoL between a child and his or her parents^20,27^. This understanding may then provide useful information for guiding clinical decision making, such as when planning rehabilitation therapy goals.

Research suggests that the parent’s own psychosocial health may be particularly impactful on their perception of their child’s quality of life^25,28^. Indeed, parents with higher perceived levels of parenting burden^20^ and distress^29^ generally provide lower ratings for their child’s HRQoL. Thus, the parent’s own HRQoL, including psychosocial factors, should also be considered when using parent- proxy reports or when investigating discrepancies between parent-proxy and child self-reports. This is particularly important for parents of children with BTHS, who may experience increased stress from dealing with the medical management of the condition, as well as emotional impact due to concerns about the child’s future and mortality^13^. Furthermore, assessing a parent’s HRQoL can help identify ways to better support the parent in caring for their child.

There have been very few studies focusing on the HRQoL of children with BTHS, though the psychosocial functioning of children with BTHS and their parents has been studied by

Storch and colleagues^13^ and Jacob and colleagues^11^. However, no prior studies have specifically focused on the agreement between child self-reports and parent proxy- reports of HRQoL in boys with BTHS. This is particularly important because research suggests that the nature and extent of discrepancies in parent-proxy reports and child self-reports of HRQoL vary depending on the condition^17^. Consequently, these factors must be investigated specifically with BTHS in order to best serve the needs of children with BTHS and their families. Therefore, the purpose of this study is to: 1) examine the agreement between child self-reports and parent proxy-reports of HRQoL for boys with BTHS and 2) investigate the relationship between parental HRQoL and parent proxy-reports of HRQoL for the child. We anticipate that parents’ own HRQoL will have positive relationships to the boys’ HRQoL as rated by the parents.

## Methods

A two-group cross-sectional design was used to investigate 1) agreement between child self-reports and parent proxy-reports of HRQoL in boys with Barth syndrome (BTHS); and 2) relationship between parental HRQoL and parent proxy-reports of HRQoL for the child. Participants were boys with BTHS, ages 4 – 17 (10.70 ± 3.70; N = 28) and one parent of each child (N = 28). Five children fell in the age group of 4 to 7, 12 children in the age 8-to-12 group, and 11 children in the age 13-to-17 group. Participants were recruited at the 2010 and/or 2012 Barth Syndrome International Conference. This conference is a scientific, medical, and family conference held in the United States every two years. The conference was attended by families from the US, Canada, United Kingdom, and France. For participants who attended both the 2010 and 2012 Conferences, only data from the 2012 Conference were used.

This study was approved by the University of Florida’s Institutional Review Board. Before data collection, written informed consent was obtained from the parent, and assent obtained from the boy. Boys with BTHS completed an HRQoL self-report, and one of the parents completed the same HRQoL instrument using the parent proxy-report version.

### Measures

#### Pediatric quality of life inventory version 4.0:

The Pediatric Quality of Life Inventory Version 4.0 (PedsQL™) was used to measure the HRQoL of boys with BTHS. Both the child report and the parent report versions were used in this study. The PedsQL™ consists of four subscales: Physical Functioning (8 items), Emotional Functioning (5 items), Social Functioning (5 items), and School Functioning (5 items)^30^. Subscale scores are calculated by scoring each item on a 5-point scale (never = 100; almost never = 75; sometimes = 50; often = 25; almost always = 0), then averaged within each subscale. The total score is the mean of all items across the four subscales with higher scores indicating better HRQoL. The reliability and validity of the PedsQL™ were established using 963 children with or without chronic illness and 1,629 parents^31^. Clinical cut points are established for the total score between 71 and 76 for children with moderate chronic conditions, and 70 or below for children with major chronic conditions^32^. The PedsQL™ subscale scores (e.g., physical functioning) and total score were used as variables in the analyses.

#### PedsQL™ family impact module:

The PedsQL™ Family Impact Module (PedsQL™ FI) was used to assess parental quality of life (QoL). The PedsQL™ FI is a 36-item questionnaire measuring the domains of Parental QoL and Family Functioning^33^. Parental QoL was used as a variable and calculated as the mean of items from the subscales: 1) Physical Functioning (6 items), 2) Emotional Functioning (5 items), 3) Social Functioning (4 items), and 4) Cognitive Functioning (5 items). In this instrument, each item is scored on a 5-point scale (never a problem = 100; almost never = 75; sometimes = 50; often = 25; almost always = 0) with higher scores representing better parental QoL. Evidence of reliability and validity of the PedsQL™ FI instrument was established with 458 parents of children or adolescents with chronic pain^34^. Statistical analysis

Distributions of the data were tested using the Shapiro-Wilk Test. Levels of agreement between child and parent reports were tested using a Two-Way Mixed-Effect Model (absolute agreement, average measures) Intraclass Correlation Coefficient (ICC). ICC value of ≤ 0.4 indicates poor-to-fair agreement, 0.41 to 0.60 means moderate agreement, 0.61 to 0.80 is good agreement, and 0.81 to 1.00 represents excellent agreement^35^. Magnitude of mean differences in child self-reports and parent proxy-reports were analyzed using paired *t*-tests. Consistency in rank order relationships between the child and parent reports were tested using the Pearson Correlation Coefficient (Pearson *r*). Spearman Correlation Coefficient (*r*_*s*_) was used to test the relationship between parental HRQoL and parent-rated HRQoL of the child. SPSS version 24 was used for all data analyses (α = 0.05; 2-tailed assumed).

## Results

All data were normally distributed as tested using the Shapiro-Wilk Test (*p* > .05). Findings from the ICC indicate that the agreement between child self-reports and parent proxy-reports was moderate-to-good. The results of the agreement in the ICC were good between the child self-reports and parent proxy-reports of HRQoL for Physical Functioning, Emotional Functioning, School Functioning, and Total Score. Moderate agreement was found in Social Functioning (Table 1).

The means of self-reports of children with BTHS were significantly higher than those of parent proxy-reports for all the scales and Total Score (*p* < .05). Magnitude of PedsQL™ mean scores were significantly different between child self-reports and parent proxy-reports in Physical Functioning (*t*(27) = 3.267, *p* = .003) , Emotional Functioning (*t*(27) = 2.319, *p* = .028), Social Functioning (*t*(27) = 3.377, *p* = .002), and School Functioning (*t*(27) = 2.559, *p* = .016), as well as Total Score (*t*(27) = 4.386, *p* < .001). Parent proxy scores were lower than child reported scores across all subscales and Total Score (Table 1). With regard to rank order of the ratings, the Pearson *r* was significant for the four subscales and total score. The child self-reports and parent proxy-reports were consistent despite differences in the magnitude of responses (i.e., ratings) (Table 1).

A positive relationship was found between parental HRQoL and parent proxy-reports of HRQoL for the child (*r* (26) = .581, *p* = .001). Thus, the higher the scores for parental HRQoL, the higher the scores for parent proxy-reports of HRQoL for the child.

## Discussion

This study investigated the extent of agreement in perceptions of HRQoL reported by boys with BTHS compared to the parent’s perception of the boy’s HRQoL. The agreement found in our study was higher than those previously reported in the literature^35,36,37,38^. In our study, we found good agreement for physical functioning, emotional functioning, and school functioning in boys with BTHS, whereas there was only moderate agreement for social functioning. Consistent with the literature, we observed higher agreement for physical functioning than for social functioning^39,40,41^. These findings suggest that parents may be more knowledgeable of their child’s physical status than of the child’s social experiences.

Children with disabilities often have constrained social participation^42^. Boys with BTHS have difficulty participating in activities requiring physical exertion, which can hinder socialization^14^. Bullying experiences related to small stature and chronic pain^13^ and potential learning difficulties and sensory issues in BTHS can also impact socially-related quality of life. Lower agreements in ratings of social functioning suggest that parents may not fully appreciate the intricacies of their child’s social experiences. Good understanding of the child’s social experiences is important for decision making when considering ways to support or facilitate the child’s social engagement. The lower agreements in ratings of social functioning (as compared to physical functioning) point to the importance of incorporating measurement of both child self-reports and parent proxy-reports of HRQoL into research and clinical practice.

In this study, parents perceived the boys’ HRQoL to be lower than that reported by the boys themselves. This is consistent with previous research in children with chronic diseases, which found that parents tend to underestimate their child’s HRQoL^35,43^. Findings may reflect parental concern regarding perceived impacts of physical difficulties on their child’s daily life and overall HRQoL. However, findings might also suggest the boys’ reduced awareness of the impacts related to their impaired health status^35^, or the presence of beneficial coping and/or compensatory strategies. Boys with BTHS grow up with physical symptoms that manifest at young ages^4^, thus creating opportunity for adjustment to physical constraints early in development. Nonetheless, the mean scores as reported by both the boys and their parents fell below clinically meaningful cutoff scores compared to moderate and major chronic health conditions (76 and 70 respectively)^32^. Therefore, our findings contribute evidence of diminished HRQoL for children growing up with BTHS.

In addition, we found a significant positive relationship between parental HRQoL and parent proxy-reports of HRQoL for the child, indicating higher parental HRQoL was significantly related to higher ratings of the parents on their children’s HRQoL. The finding is consistent with other studies that reported associations between parent proxy-report of HRQoL for the child and parental HRQoL^28^, parental stress^44^, and parental health^45^. Our finding contributes to the existing evidence that points to the importance of assessing the parent’s own HRQoL when the parent rates the child’s HRQoL.

Client-centered care concentrates on the child’s needs and functioning rather than focusing solely on the disease; such care contributes to high-quality, desirable, and effective healthcare services^46^. Assessing the child’s HRQoL provides valuable understanding for client-centered care. Quality of life is inherently subjective, and the child’s perceptions provide salient information. Therefore, children who can report on their perceived quality of life should report themselves. However, parent proxy-reports of HRQoL can provide information different from that reported by the child. Therefore, parent proxy-reports should be used in conjunction with the child self-report when making client-centered health decisions. This is because parent proxy-reports remain an important source of complementary information about the child’s HRQoL.

## Conclusion

This study broadens understanding of HRQoL of boys with BTHS using both child self-reports and parent proxy-reports. HRQoL measurement using both child self-reports and parent proxy-reports can contribute to demonstrating the value of interventions in research and clinical practice. Our sample is not large enough to generalize results. However, it is notable that according to the BTHS registry^4^, our sample size of 28 children represents almost 6% of the known population of individuals with BTHS. Sample size hindered our ability to assess potential impacts of parenting role (e.g., mother versus father) on proxy-reporting. Future research should include investigation of factors such as the child’s age, disease severity, family functioning, and family cohesion in order to elucidate child and parental factors affecting level of agreement between child self-reports and parent proxy-reports.

## Figures and Tables

**Table 1.** HRQoL mean scores and the agreement between children and parents.

	Child self-report	Parent proxy-report	*p*	CI^++^	Pearson *r*	ICC^+^
	Mean (SD)	Mean (SD)				
Physical Functioning^*^	62.28 (21.98)	49.89 (24.26)	.003	[4.60, 20.17]	.627^**^	.712
Emotional Functioning^*^	70.18 (17.13)	63.21(18.27)	.028	[.80, 13.13]	.599^**^	.720
Social Functioning^*^	70.89 (17.90)	58.11 (18.66)	.002	[5.02, 20.55]	.400^*^	.493
School Functioning^*^	66.61 (21.90)	55.71 (23.56)	.016	[2.16, 19.63]	.511^**^	.634
Total Score^*^	66.81 (17.31)	55.82 (15.96)	<.001	[5.85, 16.13]	.685^**^	.723

* *p*<0.05.

** *p*<0.01.

+ Intraclass correlation coefficient of ≤0.40 = poor-to-fair agreement, 0.41 to 0.60 = moderate agreement, 0.61 to 0.80 = good agreement, and 0.81 to 1.00 = excellent agreement^35^.

++ 95% confidence interval of the difference
